# P-1752. Antimicrobial Stewardship Initiative on Prescribing at Discharge from a Community Medical Center

**DOI:** 10.1093/ofid/ofae631.1915

**Published:** 2025-01-29

**Authors:** Gargi Adenkar, Karan Raja, Brandon Chen, Mitesh Patel, Mona Philips

**Affiliations:** CMMC, Belleville, New Jersey; Clara Maass Medical Center, Belleville, New Jersey; Clara Maass Medical Center, RWJBarnabas Health, Belleville, NJ; Clara Maass Medical Center, RWJBarnabas Health, Belleville, NJ; Clara Maass Medical Center, RWJBarnabas Health, Belleville, NJ

## Abstract

**Background:**

Inappropriate antimicrobial prescribing and overuse propagates multi-drug resistant organisms and increases risk of adverse drug events. Antimicrobial stewardship programs (ASP) focus a majority of interventions on hospital inpatients, but efforts at discharge remain limited. A recent gap analysis at our institution found that 53% of antimicrobial regimens prescribed at discharge were inappropriate in drug choice, dosing, and/or duration. Therefore, a multifaceted ASP initiative focusing on discharge antimicrobial regimens was implemented at our institution. The study objective was to assess program impact on prescription appropriateness.

Overall Appropriateness of Antimicrobial Prescriptions

**Figure:**
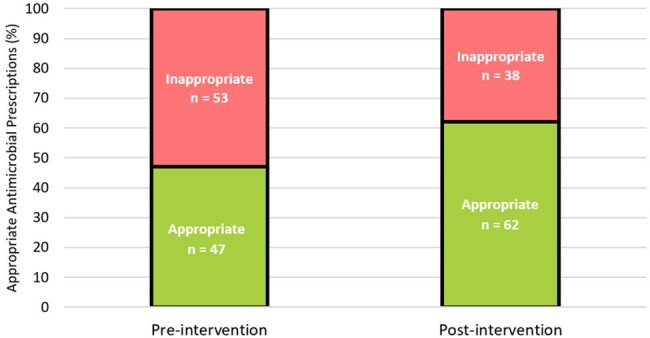

**Methods:**

Components of the stewardship initiative included development of an institution-specific prescribing guideline, clinician education, and pharmacist prospective audit and feedback at discharge. This IRB-approved study evaluated oral antimicrobial regimens prescribed to adults patients upon discharge from a community medical center. Patients were randomized irrespective of encounter type or discharge destination. Pregnant and post-partum patients were excluded. The validated National Antimicrobial Prescribing Survey (NAPS) tool was used to categorize antimicrobial appropriateness as appropriate (optimal or adequate), inappropriate (suboptimal or inadequate), or not assessable. Hospital-specific treatment guidelines, literature references, and patient-specific factors were used to determine appropriateness. The primary outcome was the proportion of appropriate discharge antimicrobial regimens before versus after initiative implementation.

Proportion of Each NAPS Category

**Figure:**
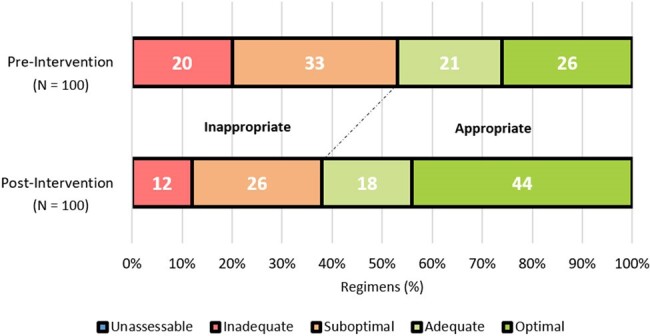

**Results:**

One hundred antimicrobial regimens were analyzed in each cohort. The proportion of appropriate antimicrobial regimens increased by 15% after program implementation (47% vs. 62%, p = 0.03). Exploratory outcome analyses indicated an increase in optimal regimens (26% vs. 44%) and a decrease in suboptimal (33% vs. 26%) and inadequate (20% vs 12%) therapy as categorized by the NAPS tool.

**Conclusion:**

ASP initiative implementation increased the proportion of appropriate discharge antimicrobial regimens. Study results highlight the positive impact of a multidisciplinary approach in improving discharge antimicrobial prescribing.

**Disclosures:**

**All Authors**: No reported disclosures

